# Distinct and Temporally Stable Assembly Mechanisms Shape Bacterial and Fungal Communities in Vineyard Soils

**DOI:** 10.1007/s00248-022-02065-x

**Published:** 2022-07-14

**Authors:** Stefano Larsen, Davide Albanese, James Stegen, Pietro Franceschi, E. Coller, Roberto Zanzotti, Claudio Ioriatti, Erika Stefani, Massimo Pindo, Alessandro Cestaro, Claudio Donati

**Affiliations:** 1 Research and Innovation Centre, Fondazione Edmund Mach, via E. Mach 1, 38010 San Michele all’Adige, Italy; 2 Pacific Northwest National Laboratory, Richland, WA USA; 3 Technology Transfer Centre, Fondazione Edmund Mach, via E. Mach 1, 38010 San Michele all’Adige, Italia

**Keywords:** Spatial scales, Beta-diversity, Null models, Neutral models, Stochasticity, Agriculture

## Abstract

Microbial communities in agricultural soils are fundamental for plant growth and in vineyard ecosystems contribute to defining regional wine quality. Managing soil microbes towards beneficial outcomes requires knowledge of how community assembly processes vary across taxonomic groups, spatial scales, and through time. However, our understanding of microbial assembly remains limited. To quantify the contributions of stochastic and deterministic processes to bacterial and fungal assembly across spatial scales and through time, we used 16 s rRNA gene and ITS sequencing in the soil of an emblematic wine-growing region of Italy.

Combining null- and neutral-modelling, we found that assembly processes were consistent through time, but bacteria and fungi were governed by different processes. At the within-vineyard scale, deterministic selection and homogenising dispersal dominated bacterial assembly, while neither selection nor dispersal had clear influence over fungal assembly. At the among-vineyard scale, the influence of dispersal limitation increased for both taxonomic groups, but its contribution was much larger for fungal communities. These null-model-based inferences were supported by neutral modelling, which estimated a dispersal rate almost two orders-of-magnitude lower for fungi than bacteria.

This indicates that while stochastic processes are important for fungal assembly, bacteria were more influenced by deterministic selection imposed by the biotic and/or abiotic environment. Managing microbes in vineyard soils could thus benefit from strategies that account for dispersal limitation of fungi and the importance of environmental conditions for bacteria. Our results are consistent with theoretical expectations whereby larger individual size and smaller populations can lead to higher levels of stochasticity.

**Supplementary Information:**

The online version contains supplementary material available at 10.1007/s00248-022-02065-x.

## Introduction

Soil microbiomes are among the most biodiverse components of all ecosystems, with diversity ranging from thousands to millions of ‘species’ in typical soil samples [1]. Recent estimates indicate that microbes represent ~ 90% of below-ground biomass globally [2], providing essential ecosystem functions and services, including carbon sequestration [3, 4] and nutrient cycling as well as directly and indirectly influencing plant and animal health, including humans [5]. The development of molecular approaches has fostered a significant leap in our understanding of soil microbiome composition and functions, revealing how they promote plant growth and increase plant resistance towards pathogens and abiotic stress [6].

Understanding the ecology of microbiomes in agricultural systems is particularly important because that knowledge can be used to manipulate soils towards beneficial outcomes. However, most studies on agricultural microbiomes focus on assessing biodiversity and community composition [7–9], rather than the assembly mechanisms that govern microbiome variation [10]. A better understanding of the mechanisms driving crop-associated microbiome assembly could be used to design new manipulation strategies based on altering both dispersal-based and selection-based processes.

Research on community assembly has emphasised a distinction between deterministic selective processes and stochastic non-selective processes [11]. The first are primarily tied to the fitness differences among species and include environmental filtering and biotic interactions such as predation and competition. The second include mechanisms that drive variation across communities independently from species differences, such as ecological drift, probabilistic dispersal, as well as local extinctions and colonisations. These two opposing processes, often referred to as ‘niche’ and ‘neutral’ respectively, operate simultaneously in ecological communities [12, 13], but their relative influences may depend on spatial scale, environmental heterogeneity, and species dispersal ability [14–16].

Studying agricultural microbiome assembly in terms of stochastic and deterministic processes provides a robust theoretical framework that has been applied broadly to microorganisms [17]. For example, the balance between stochastic and deterministic processes has been shown to vary over soil depth gradients and with the intensity of land use [18–20] as well as across spatial scales [21]. In addition to varying across physical gradients, assembly processes are also likely to vary across co-occurring microbial groups. In particular, the two dominant components of the soil microbiota, namely bacteria and fungi, display markedly different ecological traits, diversity, and dispersal ability. In turn, bacterial and fungal communities are likely influenced by distinct assembly mechanisms [20, 22].

Knowledge of the relative contributions of different assembly processes is important both for fundamental understanding and also practical management of agriculture soil microbiomes. For example, the degree to which deterministic selection or dispersal limitation influence soil microbial communities can determine the success of management strategies with respect to environmental manipulation or inoculum transplants across locations [1, 23]. Knowledge of assembly processes is particularly important for viticulture and wine production, where the fermentation process and both the quality and flavour of wine strongly depend on microbial activity from the vineyard to the winery [24]. Furthermore, the soil microbiome is considered the key natural source of grape- and must-associate microbiota [25, 26] and consistent biogeographic differences in soil and climate contribute to regional wine characteristics via the so-called ‘microbial terroir’ [8, 25]. To use knowledge of assembly processes towards viticulture (or other agricultural) applications requires theoretical coherence in our understanding such that knowledge may become transferable from one system to another. This is currently challenging in viticulture systems. For example, studies assessing microbial assembly processes in vineyard systems have highlighted that while the influence of spatial, dispersal-related processes can be significant even at local scales (2 km), the effect of deterministic niche selection can be dominant [10, 27]. In addition, regional patterns in microbial ecology can correlate with wine metabolites [28], but the main scale of variation in microbiomes appears context-dependent. The degree to which inter-vineyard variation compares to intra-vineyard variation and how spatial variation compares to temporal variation also remains unclear [8, 25, 27, 29].

Our aim here is to advance understanding of microbiome assembly processes both for fundamental understanding and for application to viticulture systems, with an emphasis on variation in assembly processes across environments, spatial scales, time, and microbial groups. We used 16S rRNA gene and ITS sequencing in soil samples across ten vineyards from four locations within Trentino, an emblematic wine-growing region in north-east Italy. We consider this system both as a model system for perennial crops within heterogeneous landscapes and as a viticulture-specific system with high regional relevance. Within each vineyard, replicates from both permanent crop areas and adjacent grasslands were sampled for two consecutive seasons. This multiscale design allowed us to examine the degree to which selective and stochastic processes influenced microbiome variation within and among vineyards and its consistency between years. To this end, we employed analytical approaches based on null-models of phylogenetic distances between communities, as well as neutral models describing the theoretical relation between taxa abundance and occurrence in the metacommunity under neutral dynamics.

A previous study on the same system found that taxonomic diversity was not correlated between bacteria and fungi [9], suggesting that distinct assembly mechanisms may be operating. Given their larger body size and smaller propagule pressure, fungal assemblages were expected to display stronger dispersal limitation and a higher degree of stochastic assembly than bacteria [20, 30, 31]*.* As a consequence, we also expected fungal communities to display stronger year-to-year dynamics. Conversely, bacteria are less dispersal-limited while also displaying high abundances. This is expected to allow bacteria to better colonise and establish populations across a range of conditions and thus increase the influence of deterministic selection.

## Materials and Methods

### Sample Collection

The sampling sites included 10 vineyards from four different locations (Ala, Besagno, Mori and S. Felice) within the Trentino Province. The study area covers c.150km^2^, over an elevation ranging 400–590 m a.s.l., and with maximum distance between locations not exceeding 15 km (Fig. S1). In each vineyard, soil samples were extracted from two land-use types, the perennial crop-covered surface (between vineyard rows, about 1 m from the vine plants) and the adjacent grassland areas at a distance of 8–16 m from the border of the vineyard. Sampling was conducted for two consecutive years (2017 and 2018) in May. For each position (each land-use type), 6 equally spaced (~ 3 m) sampling replicates were performed, for a total of 180 samples for each sampling year. The dominant grass species in crop sites were species of the Poaceae family, while grassland sites were dominated by *Arrhenatherum elatius*, *Bromus erectus* and *Trisetum flavescens*. Three different varieties are grown across the vineyards, including Chardonnay, Pinot Grigio and Müller-Thurgau. Because vineyard location had a stronger influence on microbial composition than grape variety, and different varieties were present within locations, we did not consider the effect of variety any further in our multi-scale assessment of assembly processes (Fig. S2).

Site coordinates and technical characteristics of the vineyards (planting year, previous crop) are presented in [9]. All samples had a similar range of soil abiotic variables, especially soil texture (loam, sandy clay loam, sandy loam and silty loam, Fig. S3). Quantity of soil organic matter (SOM), total nitrogen, total carbonate, and heavy metals for both vineyard and grassland samples are reported in Tab. S1 and S2. Climate is typical for the Mediterranean Alps, with warm summers and precipitation concentrated in the winter months. Main climatic variables, including air temperature and humidity, were similar across locations and are reported in Tab. S3.

Samplings were executed collecting 20 cm of soil by means of a manual, one-piece, and 7-cm diameter drill for loamy soils (Eijkelkamp, Edelman model). For chemical analysis and for taxonomic purposes the first 5 cm of soil were discarded. Each sample consisted of 4 drillings that were homogenised in a plastic bag. From each bag, a small volume of soil was collected in a 50-ml tube and chilled to 6/8 °C during the sampling time after which they were frozen at − 18 °C until sequencing.

### DNA Extraction, Library Preparation, and Sequencing

The soil samples were frozen, dried and sieved with a 0.2-mm mesh size and stored at − 80 °C until DNA extraction. The total DNA was extracted from 0.25 g of each composite soil sample using the PowerSoil DNA isolation kit (MO BIO Laboratories Inc., CA, USA) according to the manufacturer’s instructions. Total genomic DNA was amplified using primers specific to either the bacterial and archaeal 16S rRNA gene or the fungal ITS1 region. The specific bacterial primer set 515F (5’-GTGYCAGCMGCCGCGGTAA-3’) and the 806R (5’-GGACTACNVGGGTWTCTAAT-3’) was used [32] with degenerate bases as suggested [33, 34].

Although no approach based on PCR amplification is free from bias, this primer pair has been shown to guarantee good coverage of known bacterial and archaeal taxa. For simplicity, in the text, we refer to these groups as bacteria to distinguish them from fungi. In the figures, however, we specify ‘Bacteria & Archea’. For the identification of fungi, the internal transcribed spacer 1 (ITS1) was amplified using the primer ITS1F (5’-CTTGGTCATTTAGAGGAAGTAA-3’) [35] and ITS2 (5’-GCTGCGTTCTTCATCGATGC-3’) [36]. All the primers included the specific overhang Illumina adapters for the amplicon library construction.

For the 16S V4 region, each sample was amplified by PCR using a 25-µl reaction with 1 µM of each primer. More in detail, 12.5 µl of 2 × KAPA HiFi HotStart ReadyMix and 10 ul forward and reverse primers were used in combination with 2.5 µl of template DNA (5–20 ng/µl). PCR reactions were executed by GeneAmp PCR System 9700 (Thermo Fisher Scientific) and the following cycling conditions: initial denaturation step at 95 °C for 5 min (one cycle); 28 cycles at 95 °C for 30 s, 55 °C for 30 s, 72 °C for 30 s; final extension step at 72 °C for 5 min (1 cycle).

For the ITS1 region, each sample was amplified by PCR using a 25-ul reaction with 10 µM of each primer. More in detail, 22 µl of premix FastStart High Fidelity PCR System (Roche) and 2 µl forward and reverse primers were used in combination with 1 µl of template DNA (5–20 ng/µl). PCR reactions were executed by GeneAmp PCR System 9700 (Thermo Fisher Scientific) and the following cycling conditions: initial denaturation step at 95 °C for 3 min (one cycle); 30 cycles at 95 °C for 20 s, 50 °C for 45 s, 72 °C for 90 s; and final extension step at 72 °C for 10 min (1 cycle).

The amplification products were checked on 1.5% agarose gel and purified using the Agencourt AMPure XP system (Beckman Coulter, Brea, CA, USA), following the manufacturer’s instructions. Afterward, a second PCR was used to apply dual indices and Illumina sequencing adapters Nextera XT Index Primer (Illumina), by 7 cycles PCR (16S Metagenomic Sequencing Library Preparation, Illumina). The amplicon libraries were purified using Agencourtusing the Agencourt AMPure XP system (Beckman), and the quality control was performed on a Tapestation 2200 platform (Agilent Technologies, Santa Clara, CA, USA). Finally, all barcoded libraries were pooled in an equimolar way and sequenced on an Illumina® MiSeq (PE300) platform (MiSeq Control Software 2.5.0.5 and Real-Time Analysis software 1.18.54.0).

Soil samples from 2017 and 2018 were sequenced in different times in several sequencing runs. To estimate potential batch effects, we resequenced 10 samples from 2017 and 10 from 2018, one for each vineyard, in a single sequencing run (‘control run’). Compositional distances between these control runs were always much smaller than the minimum distance between adjacent sample replicates, indicating that any batch effect is unlikely to influence our results and conclusions (Fig. S4).

### Bioinformatic Processing

The sequences were assigned to samples using sample-specific barcodes and saved in FASTQ-formatted files. Raw data FASTQ files were analysed using the software pipeline MICCA [37] v. 1.7.2.

Raw overlapping 16S paired-end reads were assembled (merged) using the procedure described in [38]. Paired-end reads with an overlap length smaller than 200 bp and with more than 50 mismatches were discarded. After trimming forward and reverse primers, merged reads shorter than 250 bp and with an expected error rate higher than 0.5% were removed.

Filtered sequences were clustered into sequence variants (SVs) using the UNOISE3 denoising algorithm available in MICCA. OTUs were taxonomically classified using the Ribosomal Database Project (RDP) Classifier [39] v2.11. Multiple sequence alignment (MSA) was performed on the denoised reads applying the Nearest Alignment Space Termination (NAST) [37, 40] algorithm and the phylogenetic tree was inferred using FastTree [41] v2.1.8.

Raw overlapping ITS paired-end reads were merged and merged sequences with an overlap length smaller than 100 bp and with more than 32 mismatches were discarded. After primers trimming, merged reads shorter than 150 bp and with an expected error rate higher than 0.5% were removed. Filtered sequences were clustered into SVs using the UNOISE3 denoising algorithm and SVs were taxonomically classified using the RDP Classifier v2.11 and the UNITE [42] database. To compensate for different sequencing depths, samples were rarefied to an even depth of 15,000 reads for both 16S and ITS sequences. Samples with less than the minimum number of reads were discarded.

### Statistical Analysis

#### Abiotic Variables

The main spatial and temporal patterns in soil abiotic variables were visualised using Principal Component Analysis (PCA). The BIOENV procedure [43] was used to subset the combination of soil variables whose Euclidean distances showed maximum (rank) correlation with community dissimilarity each year (Bray–Curtis distance). The influence of these variables on microbial composition was further examined and visualised with distance-based redundancy analysis (db-RDA; [44]. These exploratory analyses were primarily used to examine the consistency of environmental effects across years. A detailed assessment of the effects of soil properties on microbial diversity is given in Coller et al. [9].

#### Diversity

Microbial α-diversity was expressed as taxonomic richness (number of SVs observed after rarefaction) and Hill-Simpson diversity of order *q* = 2, which emphasises the contribution of abundant taxa [45].

Compositional variation between samples was expressed as Bray–Curtis dissimilarity using log-transformed abundances. Variation was then compared over multiple spatial scales, that is, between local replicates, land-uses (i.e., cropland vs grassland), vineyards and locations. Dissimilarity between years was also quantified. Each comparison was calculated by first fixing the other scale. For instance, variation among replicates only included samples within the same land-use, same year, vineyard and location. Similarly, variation among vineyards included samples within the same year, land-uses and location. Therefore, each comparison exclusively quantified compositional variation at one spatio-temporal scale.

To further quantify year-to-year changes in composition, we calculated the shift in rank position between years for each taxa (for taxa observed in both years) using the rank_shift function from codyn package [46]. Rank shift was then expressed as percentage change.

#### Null-Model of Phylogenetic and Taxonomic β-diversities

To estimate the contribution of different assembly mechanisms to metacommunity assembly, we used the null-modelling framework originally developed by Stegen et al. [47]. The approach integrates abundance-based null-models of phylogenetic and taxonomic β-diversities to classify pairs of assemblages based on the contribution of deterministic and stochastic processes. Phylogenetic β-diversity was calculated as the β-NTI (beta nearest-taxon index). This index quantifies the degree to which the β-mean-nearest taxon distance (β-MNTD) deviates from null expectations based on 999 random shuffles of SVs across the tip of the phylogenetic tree. Therefore, the β-NTI controls for the observed taxonomic β-diversity and was used to determine whether communities were shaped by deterministic or stochastic processes. In particular, values of |β-NTI|> 2 indicates deterministic selection which is then partitioned into variable (β-NTI > 2,communities phylogenetically more dissimilar than expected) and homogenous (β-NTI < 2; phylogenetically more similar) selection. The remaining community pairs with |β-NTI|< 2 were considered primarily regulated by stochastic processes. Raup-Crick-based Bray–Curtis (RC_bray_) was then used to further classify the stochastic fraction. Briefly, RC_bray_ was calculated by reassembling local communities probabilistically, accounting for the relative abundance and occurrence of each SVs. The procedure was repeated 999 times, while maintaining the observed richness and number of counts in each community. From these 999 simulations, the null distribution of Bray–Curtis distance was derived for each community pair; finally, the distance between the empirically observed Bray–Curtis and null distribution was standardised between − 1 and 1 [47, 48]. Values of RC_bray_ < 0.95 indicate communities influenced by homogenising dispersal (taxonomically more similar than expected), while dispersal limitation (combined with drift) results in RC_bray_ > 0.95 (taxonomically less similar than expected). Values of |RC_bray_|< 0.95 indicates that no particular assembly process dominates (defined as undominated). The null models were run using the algorithms implemented in the iCAMP package [49]. In line with previous works, and for computational purposes, we included the 3000 most abundant bacterial and fungal SVs in the null-modelling framework [47, 50].

Assessing community assembly using phylogenetic null-models assumes that phylogenetic distance between species reflects their niche differences (i.e. phylogenetic signal). We examined phylogenetic signals by plotting mantel correlograms between species phylogenetic distances and niche distances based on different soil physico-chemical parameters. Niche distances between species were expressed as the absolute difference in niche value, defined as the abundance-weighted mean of the environmental parameters where the species was present [51].

#### Neutral Model

To further appraise the overall importance of neutral dynamics in structuring bacterial and fungal communities, we used Sloan’s modelling framework, which assumes no fitness differences among taxa and ignores their phylogenetic relationships, but seeks to fit SVs frequency of occurrence as a function of their relative abundance in the metacommunity. The probability density function that describes the theoretical relationship between taxa abundance and occurrence has the form of a beta distribution with a single-free parameter [52]. This parameter, called *m*, represents an estimate of OTUs migration rate within the metacommunity. It thus reflects the probability that a loss of one cell (an individual) in a local community is replaced through dispersal from the metacommunity. Lower *m* values indicates higher dispersal limitation. The fit of Sloan’s neutral model was compared to that obtained from a binomial model, which represents the case where local communities are a random subset of the metacommunity in the absence of dispersal limitation [52, 53]. The neutral model was fitted using the snm function in the iCAMP package.

#### Nice Breadth

We also calculated species niche breadth using Levin’s index [54] via the niche.width function in the spaa package:

$${B}_{j}=1/\sum {p}_{j}^{2}$$

where *B*_*j*_ is the niche breadth value of species *j* and *p*_*j*_ is the proportion of species *j* in each community. Levin’s niche breadth does not explicitly include environmental parameters and represents a measure of the diversity of communities in which the species is observed.

## Results

After preprocessing and filtering, we obtained 10,924,706 16S V4 and 14,672,492 ITS1 sequences, which were denoised into 30,869 sequence variants (SVs) for bacteria and 14,099 SVs for fungi. The samples were rarified to 15,000 reads per samples both for bacteria and fungi, obtaining a dataset of 5,220,000 reads and 30,815 SVs from 348 samples for 16S, and 5,115,000 reads and 14,021 SVs from 341 samples for ITS. After excluding samples for which only 1 year of data was available, the final dataset included 308 samples from both bacteria and fungi.

### No Change in Soil Parameters Between Years

The first two axes of the PCA of soil abiotic variables accounted for more than 50% of variation (Fig. S5). The first axis represented a gradient of soil organic and nitrogen content, while the second axis separated sandy soils from wetter soils rich in silt. Although 2018 was more wet than 2017 in terms of accumulated rain (Fig. S6), no apparent changes were evident in soil physical–chemical properties across years (permanova *R*^2^ = 0.0003; NS).

As observed in previous works, soil chemistry exerted the strongest influence on both fungal and bacterial composition. The BIOENV procedure identified, for both years, the most important variables, which explained less than 10% of compositional variation in both taxonomic groups according to db-RDA (Fig. S7). For bacteria, the five most important variables consistently included silt and clay content, pH, Cu and Pb. For fungi, selected variables changed slightly between years, but similarly included silt, clay and organic matter content, pH, Cu and Pb.

### Bacteria Display Higher α-Diversity than Fungi and more Stable Composition Between Years

**Fig. 1:**
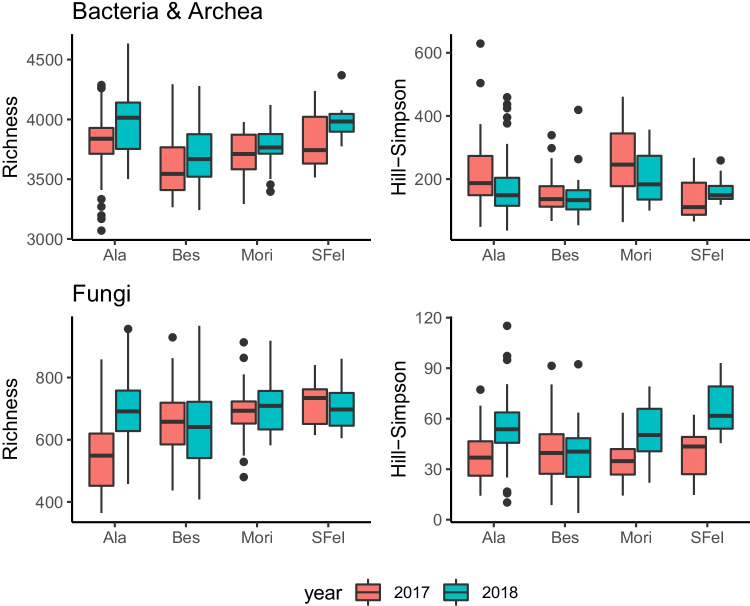
Taxonomic richness and Hill-Simpson diversity (Hill diversity of order *q* = 2) for bacteria & archea and fungi over the 2 years of study in the four vineyard locations (Ala, Besenello, Mori, San Felice)

Overall richness was almost an order of magnitude higher for bacteria than for fungi. Bacterial richness increases slightly in 2018 in all locations, although this was mostly due to an increase in rare species, as indicated by the concomitant decline in Hill-Simpson diversity (Fig. 1). Conversely, while fungal richness appeared relatively stable across years, apart from an evident increase in the location of Ala, Hill-Simpson diversity increased substantially in most locations, indicating an increase in the proportion of locally common taxa in 2018.

The overall shift in the relative abundance between years was larger for fungi than bacteria, with a mean rank shift (see the ‘Methods’ section) of taxa of about 20% compared to 11% for bacteria. This is visible in Fig. S8 where the relative abundance of the same genera is compared across each sample between years, showing a larger scatter for fungi.

The two groups also showed distinct temporal turnover dynamics. In particular, temporal turnover in fungal assemblages involved taxa with relatively high local abundances i.e. taxa that in a given location were only observed in one of the 2 years showed relatively high abundances, similar to the abundance of core taxa present in both years. This was in contrast to what was observed in bacteria, where taxa unique to a given year were relatively rare (Fig. S9).

### Little Coherence in Beta-diversity Between Bacteria and Fungi

We then examined compositional variation in bacterial and fungal communities between sample pairs across the spatial and temporal scales of the study, namely between replicates, land-uses, vineyards, locations and between years. Each comparison was calculated by first fixing the other scales (see the ‘Methods’ section).

**Fig. 2:**
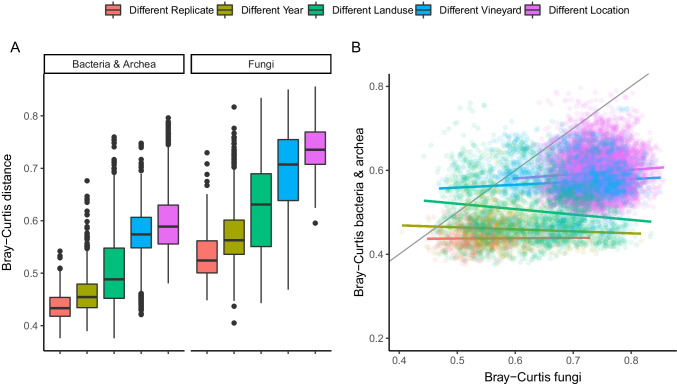
**A** Pairwise Bray–Curtis distance between samples for bacteria & archea and fungi across the scales of the study (i.e. between local replicates, years, land use, vineyards and locations). Each comparison is calculated by first ‘fixing’ the other scales. **B** Relationship between fungal and bacterial pairwise Bray–Curtis distances for each scale of the study. The thin grey line shows the hypothetical 1:1 relationship

Overall, bacteria and fungi displayed similar scaling patterns, although compositional variation between pairs of samples was consistently higher for fungi (Fig. 2A). Patterns were similar when compositional variation was based on Jaccard incidence data, indicating that effects were not only due to differences in relative abundances (Fig. S10). As expected, the smallest variation was observed between adjacent replicates within vineyards and land-use type, immediately followed by variation between years. This indicates that temporal variation in composition was only slightly larger than variation among local replicates. Compositional variation increased progressively when soil communities were compared across different land-uses, different vineyards and locations, in line with the increasing spatial scale.

However, we observed little coherence in pairwise variation between bacteria and fungi (Fig. 2B). That is, for a given comparison between samples, large compositional variation in bacterial communities was not paralleled by similarly large variation in fungi, and vice-versa. Coherence was especially low at smaller spatial scales and increased slightly at the larger, among-location scales (i.e. relationship became increasingly positive). This suggests that the two taxonomic groups were likely regulated by distinct assembly processes.

### Bacteria and Fungi Show Distinct but Temporally Consistent Assembly Mechanisms

We estimated the contribution of different mechanisms to the assembly of the soil microbiome and to what extent these mechanisms could explain the higher taxonomic variability of fungal communities relative to bacteria.

We used Mantel correlograms to measure the correlations between phylogenetic distance and niche distances based on soil abiotic variables (Fig. S11). The correlation was significant for several soil physical and chemical properties, and the signal was stronger at short phylogenetic distances, as also observed elsewhere [47, 55, 56]. This suggested quantifying phylogenetic turnover would be most robust using β-NTI (beta nearest-taxon index), a metric based on distances among close relatives. To estimate the contribution of different mechanisms to metacommunity assembly at different spatial scales, we used a modelling framework that integrates abundance-based null-models of phylogenetic β-diversities (standardised β-NTI) and taxonomic β-diversities (Bray–Curtis based Raup-Crick dissimilarity; RC_bray_). Using this approach, the observed differences between pairs of samples across spatial and temporal scales were classified as dominated by *selection* (variable or homogenous), *dispersal limitation* (combined with drift), *homogenising dispersal* and *undominated* (see the ‘Methods’ section).

**Fig. 3:**
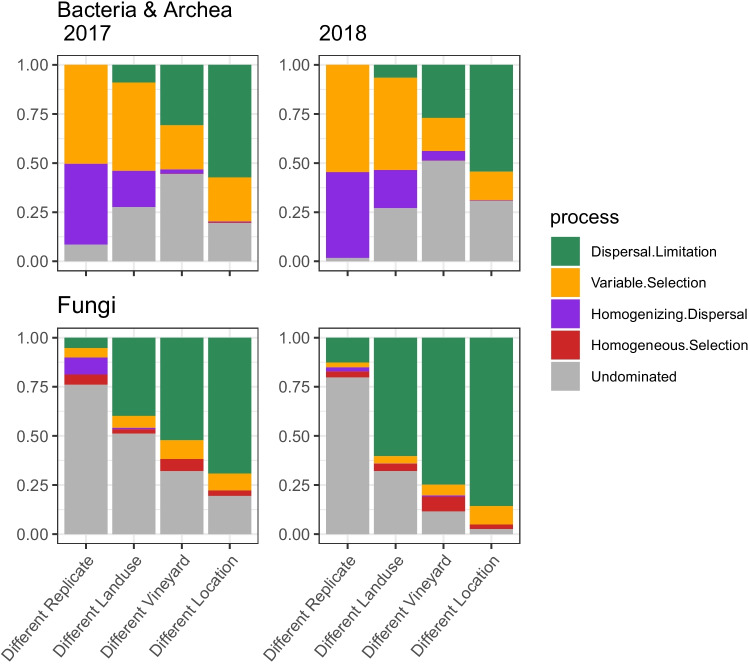
Contribution of different assembly processes across the spatial scales of the study. The relative values of assembly processes reflect the proportion of pairwise comparisons dominated by the given process as inferred from the taxonomic- and phylogenetic-based null-modelling

Overall, we found that bacterial and fungal communities were indeed regulated by different assembly processes, which were consistent over the 2 years of observation. However, the two taxonomic groups also showed some coherent responses to the varying spatial scale (Fig. 3). For bacteria, homogenising dispersal and variable selection dominated at the small between-replicates scale, while the importance of dispersal limitation increased and became prevalent at the larger between-locations scale. Similarly, the influence of dispersal limitation also grew with increasing spatial scale for fungi, although its overall contribution was clearly stronger than for bacteria at all scales. No particular assembly process dominated fungal assembly at the between-replicates scale. In both taxonomic groups the contribution of homogenising dispersal declined with increasing scale of comparison, albeit this was far more evident for bacteria. Overall, the contribution of stochastic processes was larger for fungi (ranging 84–94% across scales) than for bacteria (ranging 45–85%).

We further estimated the overall contribution of neutral dynamics to microbial assembly using Sloan’s neutral modelling framework, which assumes that each local community is in contact with a reservoir from which taxa can immigrate with a single taxon- and site-independent migration rate *m*. In this framework, a lower value of *m* corresponds to a higher impact of dispersal limitations on community assembly. A value of *m* = 1 implies that each local community is simply a random sampling from the overall metacommunity.

**Fig. 4:**
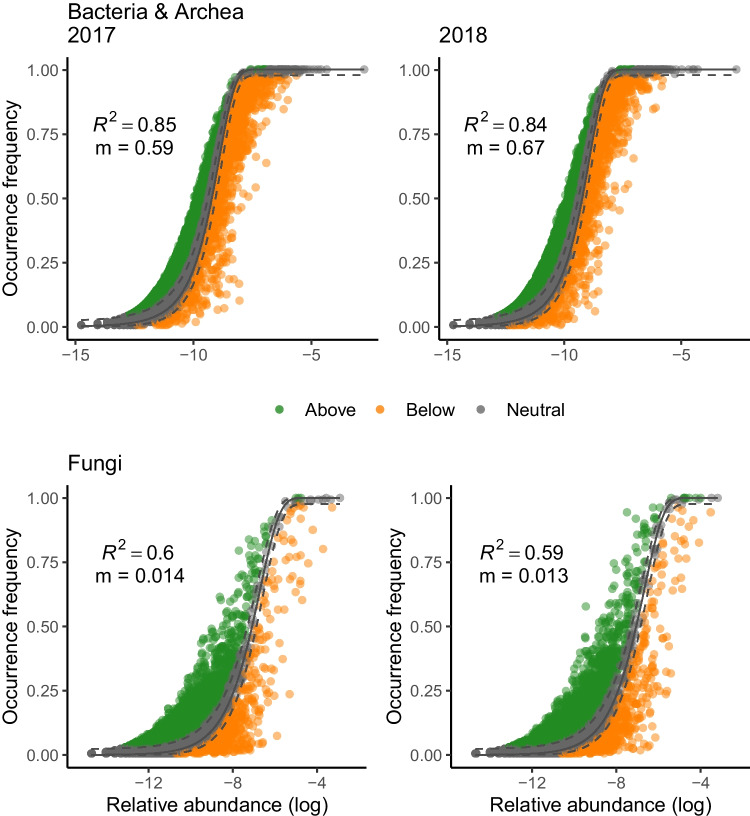
Fits of neutral models relating SVs relative abundance with their occurrence frequency across the metacommunity for bacteria & archea and fungi. Solid and dashed lines indicate model fit and 95% CI, respectively. Colours indicate SVs whose occurrence was either below or above model expectations. Model *R*-square and estimated migration rate (m) are shown

We found that the Sloan neutral model provided a good description of the data for both taxonomic groups (Fig. 4), and the quality of the fit was higher for bacteria (*R*^2^ = 0.85–0.84) than for fungi (*R*^2^ = 0.59–0.60). The estimated value of *m* was much higher for bacteria than for fungi (Bacteria = 0.59–0.67; fungi = 0.013–0.014), indicating a stronger effect of dispersal limitations for the latter. Compared to a binomial model (which assumes that communities are random subsets of the metacommunity in the absence of dispersal limitation), the Sloan neutral model was more strongly supported for fungal than for bacterial communities (Tab. S4), as expected from the lower value of *m*.

Although spatial scales are ignored in this framework, the lower quality of the fit to the Sloan model is consistent with our previous result that dispersal limitations dominate fungal community assembly at the larger spatial scale. In this scenario, we can expect fungi to display higher heterogeneity in metacommunity composition, thus violating the assumptions of neutral dynamics. Consistently with this interpretation, we also found that Levin’s niche breadth was larger for bacteria (mean = 4.98 ∓ 0.48) than for fungi (mean = 2.83 ∓ 1.12), indicating that bacterial species were, on average, more widely distributed across the study area.

## Discussion

Our understanding of the diversity and assembly of soil microbial communities has grown steadily in recent years, thanks to improved molecular and modelling techniques [57, 58]. Applications to agriculture are also increasing, with a progressive shift from single specific microbes to holistic community-level approaches [1]. However, examinations of the assembly mechanisms regulating microbial biodiversity and composition remain rare, especially in crop ecosystems.

We demonstrated that the two main taxonomic groups of vineyard soil microbiomes are regulated by distinct assembly processes over a range of spatial scales. In line with our expectations, fungal metacommunities displayed stronger dispersal limitations and were dominated by non-selective processes, whereas deterministic selection played a stronger role in bacterial assembly. In both groups, the influence of dispersal limitation clearly increased with spatial scale, from within to between vineyards and locations. These patterns were consistent over the 2 years of observation.

In line with recent investigations of soil microorganisms across a range of ecosystem types, we have shown that bacterial and fungal communities in vineyard soils display assembly patterns that are consistent with their known ecology and body size [18, 20, 30, 31]. These findings have both fundamental and applied implications.

The balance between stochastic and deterministic processes in metacommunities is known to be mediated by a suite of biotic and abiotic factors [14, 16, 59, 60]. In particular, the strength of selection appears to vary with organism size and niche breadth, but with effects depending on ecosystem types and spatial scales. While some studies reported stronger determinism for larger organisms with narrower niches [19, 61], our results parallel recent analyses in showing that the influence of deterministic selection is stronger in bacterial communities with smaller cell sizes and higher dispersal rates compared to fungal communities [20, 31, 62]. The rapid growth rates and flexible metabolism may also allow bacterial taxa to colonise diverse habitats and establish successfully, as indicated by their broader ecological niche [63, 64]. This would allow bacteria to better ‘track’ suitable environments, thus increasing the influence of deterministic selection. Conversely, fungal cells are generally less abundant and more prone to stochastic demography and local extinctions [31, 65]. This is also reflected in the larger fluctuations that we observed in the abundance of fungal taxa between years. In addition, and in line with other studies in both natural and managed ecosystems, fungal populations in soils displayed limited dispersal capacity [20, 22, 31]. In agreement with theoretical predictions, limited dispersal ability and smaller population sizes likely combined to increase the influence of stochasticity and drift on fungal dynamics [66].

Importantly, these conclusions were supported by two independent but complementary modelling approaches. First, phylogenetic null-models revealed the consistently larger influence of dispersal limitation for fungi. Second, and in line with the first, parameters from neutral model estimated fungal migration rates that were more than one order of magnitude lower than bacteria. The two modelling approaches are seldom used in combination [31, 55, 64], but our findings indicate that they could complement each other and further support conclusions regarding the importance of dispersal in metacommunities. Estimates of assembly processes from null-models require some level of phylogenetic signal in the communities and results may be sensitive to randomisation algorithms, similarity metrics and species pool, and should be interpreted on a relative basis [62]. Sloan’s neutral model, conversely, is an analytical description of the theoretical abundance-occurrence relationships in metacommunities as a function of *m,* a taxon-independent migration rate.

It must be noted that multiple approaches have been used previously to estimate the relative influence of niche-related and spatial processes in natural metacommunities. These include variation partitioning of spatial and environmental factors (e.g. [22, 64], distance-decay analysis (e.g. [20] and null-model partitioning of *α* and *β*-diversities [10]. While no method is inherently superior, we have here employed two strategies specifically developed for analysing large microbial assemblages. Concordance between the outcome of the two approaches thus provides further confidence in the results.

Although the influence of spatial scale on metacommunity assembly is well documented [60, 67, 68], this study is among the first to estimate changes in assembly processes across scales using taxonomic and phylogenetic null-models. Notably, the importance of dispersal limitation grew coherently with increasing spatial scales for both bacteria and fungi, while the influence of homogenising dispersal declined. Results indicate that scaling is an important aspect of microbial assembly in vineyard soils even within a relatively small study area, such as the one considered here (< 150 km^2^). This provides further support for the existence of small-scale biogeography of vineyard-associated microbes, especially fungi, which can contribute to defining the *microbial terroir* [27, 69]*.*

One interesting and unexpected pattern that emerged was the relatively stronger role of variable selection for bacteria at small scales (between replicates and land use in the same vineyard), relative to larger scales. This indicates that, after accounting for the limited taxonomic turnover between nearby samples, bacterial communities were phylogenetically more distant than expected, thus manifesting the imprint of deterministic selection. This is despite adjacent replicate samples being more similar than samples from different locations, according to the measured variables. The available data do not allow a deeper understanding of the mechanisms involved, but they could include the effects of unmeasured soil variables (e.g. micro-scale spatial structure) and biotic interactions (e.g. competition with fungi). Both are possible and warrant further exploration. For instance, if micro-scale differences among samples supported a few phylogenetically distinct taxa, such phylogenetic turnover would have a significant influence on β-MNTD at short spatial scales where taxonomic similarity was higher. At larger scales, however, increasing taxonomic differences would progressively ‘dilute’ the influence of phylogenetically distinct taxa, thereby reducing the signal of variable selection.

More generally, this study also highlights the importance of taxonomic and phylogenetic null models in studying metacommunity assembly. Had we relied exclusively on observed patterns in alpha- and beta-diversity, the key distinctions between fungal and bacterial assembly would not have emerged as clearly. Nonetheless, the limited coherence in pairwise β-diversity between the two taxonomic groups (Fig. 2B) suggested that different mechanisms were likely involved. This lack of coherence in spatial turnover between fungi and bacteria was also observed in Powell et al. [20], who emphasised the importance of stochasticity and priority effects in fungal assembly.

Although we focused on the soil microbiome of vineyards, increasing evidence suggests that wineyard and surrounding soil is a key source of grape-associated microbiota [26, 70]. Although the pathways by which microbial processes in soil influence wine flavours remain unclear, soil microbial communities may ultimately link vineyards’ geology and soil type to the organoleptic property of the wine produced.

Among all agricultural products, wine shows undoubtedly the strongest geographic patterns [71], and studies like ours help understand how distinct microbial signatures are established and maintained across managed landscapes. Our findings suggest that dispersal limitation may underpin geographic patterns in fungal assemblages even at small spatial scales, and thus contribute to the microbial aspects of terroir. At the same time, however, fungal assemblages displayed relatively high compositional variation across time and space and a weak contribution of deterministic selection relative to stochastic processes. This implies that predicting ecological responses to environmental change and successfully implementing management actions may be challenging.

Similarly, the temporal stability of the microbial signature in vineyards is fundamental to maintaining regional specificity. Perhaps the degree to which vineyard microbial communities vary across years may have far greater implications for the concept of microbial terroir than variation across communities within years. Studies that explicitly assess microbiome variation through time remain rare, but are indispensable to properly estimate ‘baseline’ temporal dynamics [72, 73]. Although only 2 years of observations were available in this study, there was higher temporal variation in fungal communities in terms of relative abundance and turnover of taxa. Our data suggest that bacteria and fungi differ in their inter-annual dynamics, which may contribute to variation in wine characteristics. However, the degree to which soil microbiome dynamics influence year-to-year wine properties remains uncertain and represents a fruitful avenue for future research [24].

Further research is clearly needed to appraise whether our results can be generalised to other crop systems. However, our findings suggest that approaches to modify soil microbiomes should account for dispersal limitation in fungal metacommunities. If desired, inoculum transplants could be used to overcome such limitations. Conversely, spatial separation—even at relatively small scales—could favour the persistence of regional heterogeneity in fungal composition if this is considered key for wine properties. Similarly, knowing where and at which scale deterministic processes are important could help design effective environmental manipulations, while also aiding predictions about year-to-year variation in the microbiome and its response to environmental changes.

## Supplementary Information

Below is the link to the electronic supplementary material.

Supplementary file1 (DOCX 2.04 MB)

## Data Availability

Raw sequencing data along with geographical and physico-chemical information are available at the European Nucleotide Archive (https://www.ebi.ac.uk/ena) under the study id PRJEB31356.
