# Supplementary material for: Distinct and Temporally Stable Assembly Mechanisms Shape Bacterial and Fungal Communities in Vineyard Soils
Source: Microb Ecol. 2022 Jul 14;86(1):337–49. doi: 10.1007/s00248-022-02065-x (PMC10293400; doi:10.1007/s00248-022-02065-x)
Supplement: Supplementary file 1 — Supplementary file1 (DOCX 2.04 MB) [file 248_2022_2065_MOESM1_ESM.docx]

**Supplementary Material**

**Distinct and temporally stable assembly mechanisms shape bacterial and fungal communities in vineyard soils**

*Stefano Larsen; Davide Albanese; James Stegen; Pietro Franceschi; E. Coller; Roberto Zanzotti; Claudio Ioriatti; Erika Stefani; Massimo Pindo; Alessandro Cestaro; Claudio Donati*

**Tab. S1** - Mean and standard deviation of the main physico-chemical properties of the soil in the vineyard samples


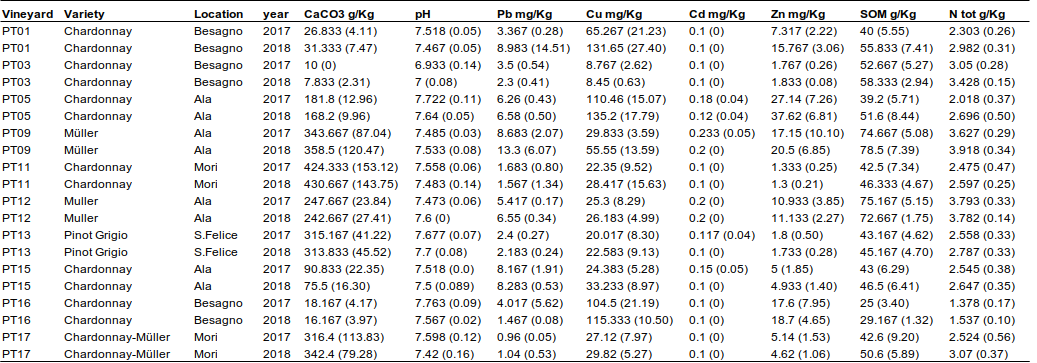


**Tab. S2** - Mean and standard deviation of the main physico-chemical properties of the soil in the grassland samples


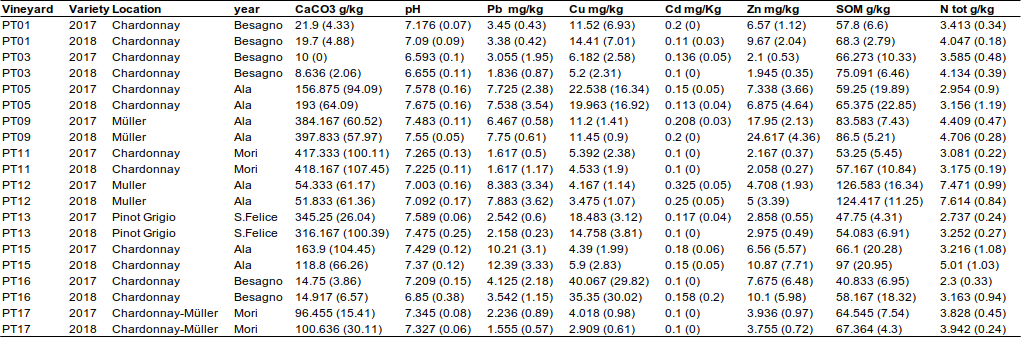


**Tab. S3** – Climatic variables for the ten study vineyards in the two years of observation


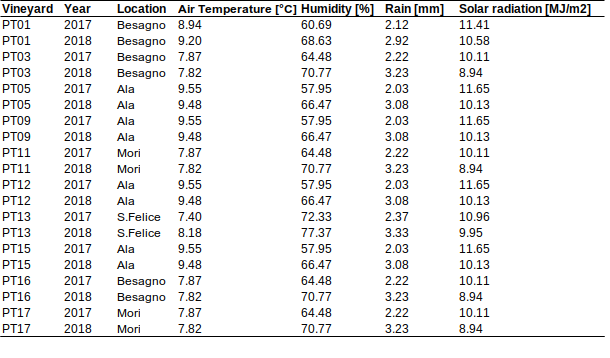


**Tab. S4** - Parameters from the neutral and binomial model fitting SVs occurrence based on relative abundance in the metacommunity.


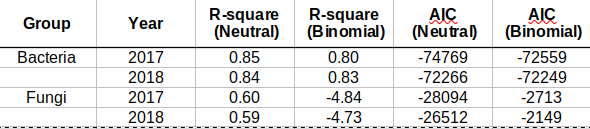


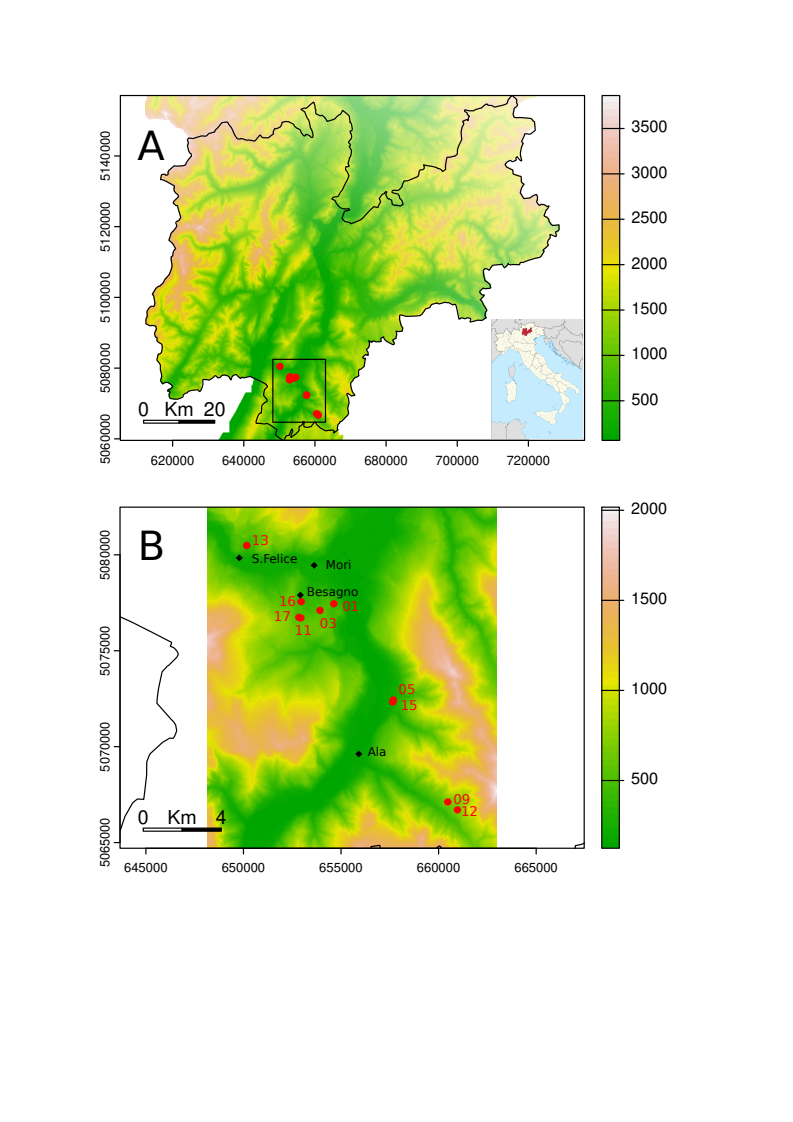
**Fig. S1** – Map of the study area, showing the ten vineyards (red dots with code number) within the Trentino Province, north-east Italy (A), and with respect to the four locations (B).

*
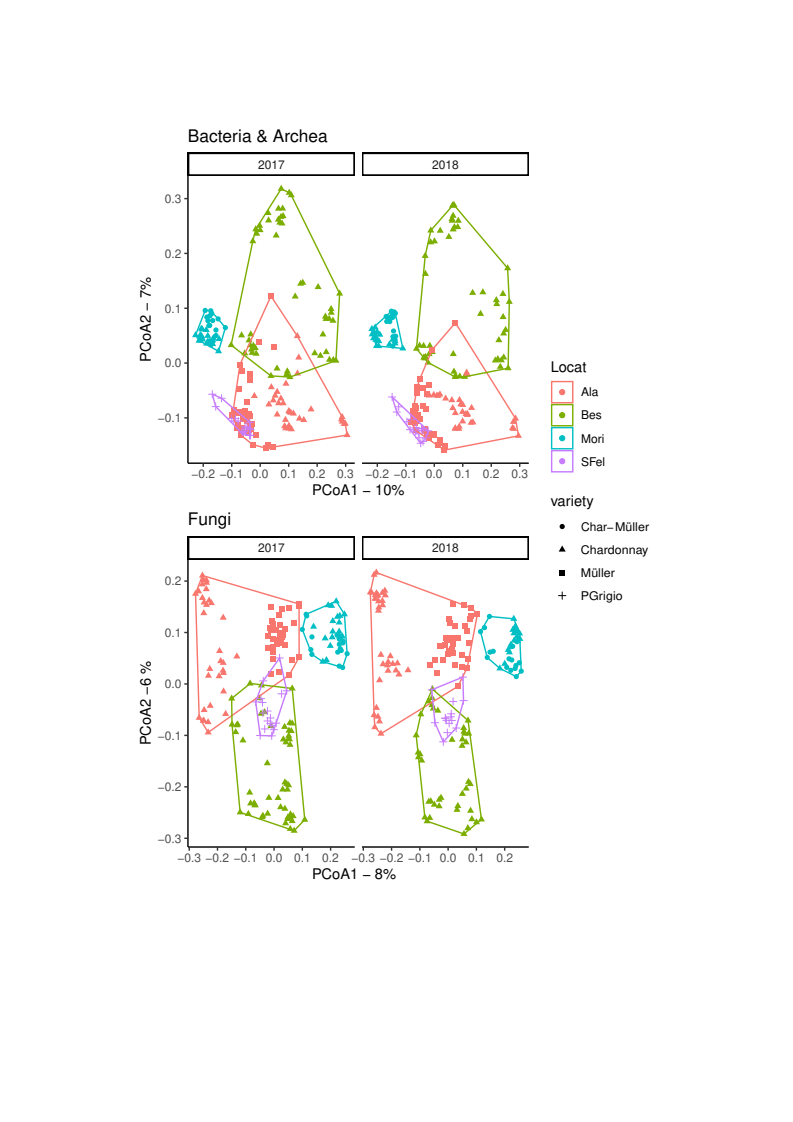
*

**Fig. S2** – PcoA plane based on the Bray-Curtis distances of log-transformed SVs abundances for Bacteria & Archea and Fungi. Locations and wine variety are shown.


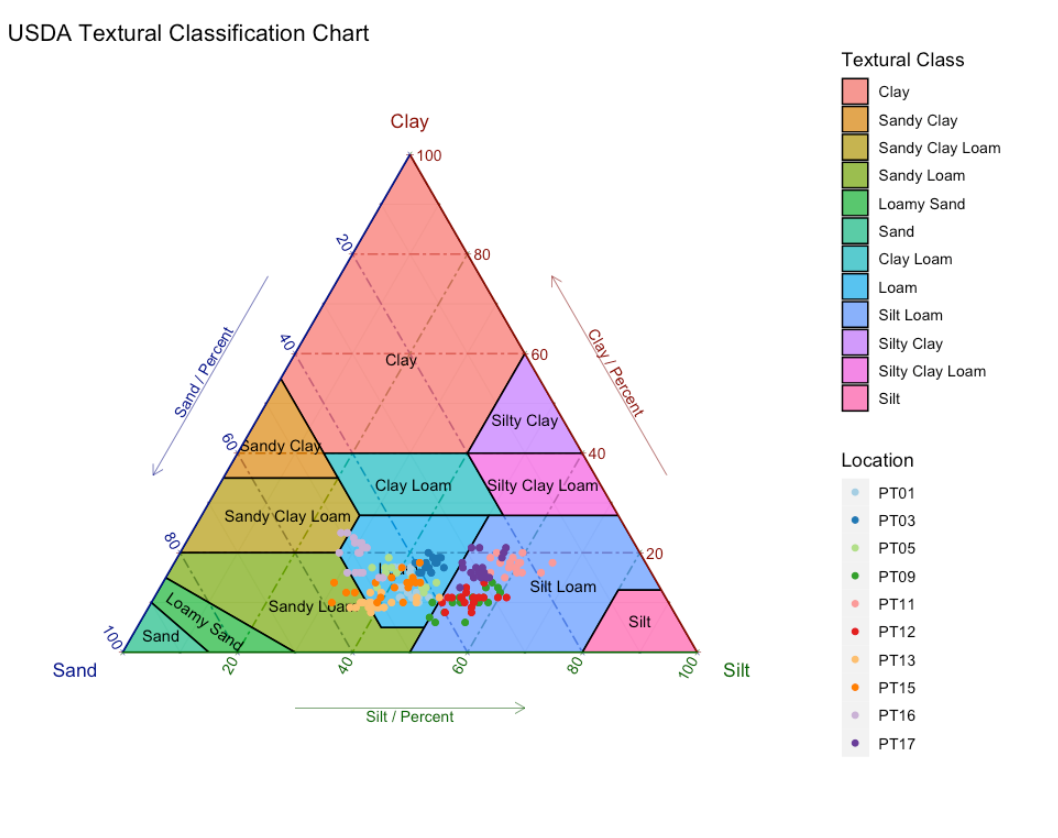


**Fig. S3 -** USDA textural classification of the analysed soil samples


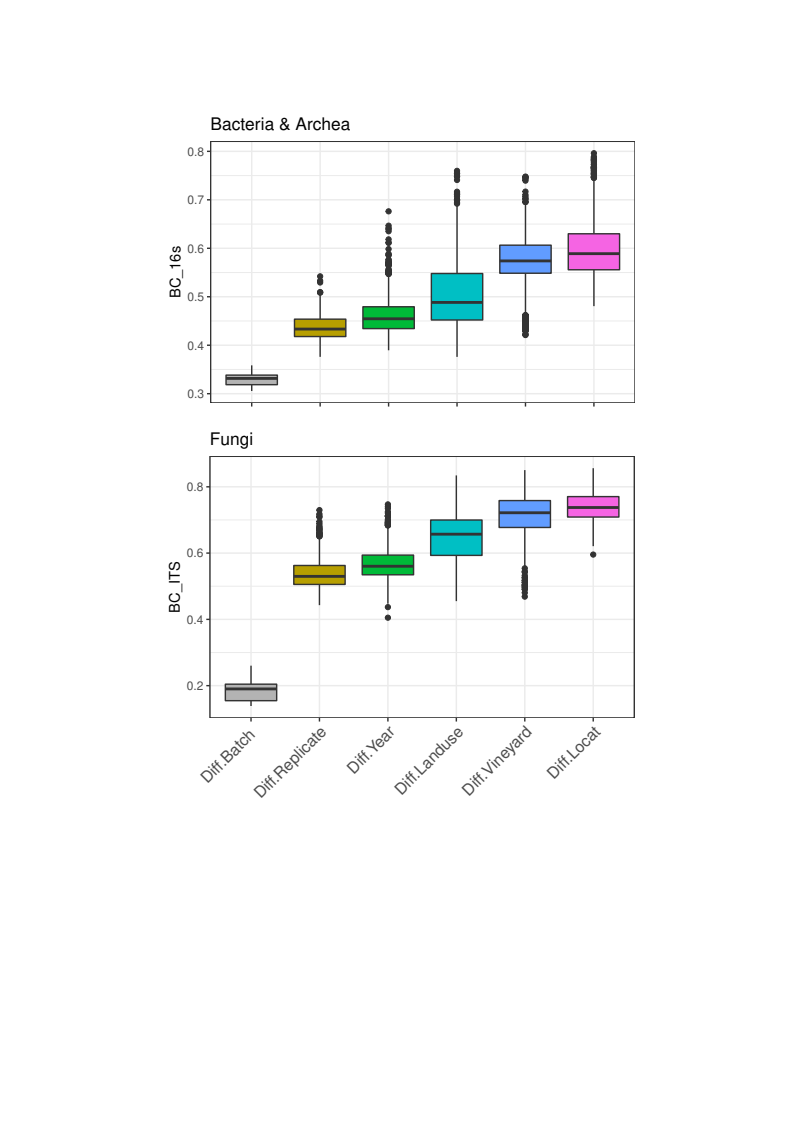
**Fig. S4** – Pairwise Bray-Curtis distance between samples across the scales of the study, in comparison to distances between different sequencing runs of the same sample (‘Diff.Batche’). Batch effect was estimated by re-sequencing 10 samples from 2017 and 10 from 2018, one for each location, in a single sequencing run.

| 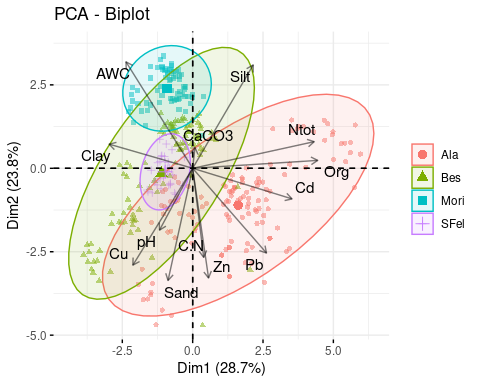 | 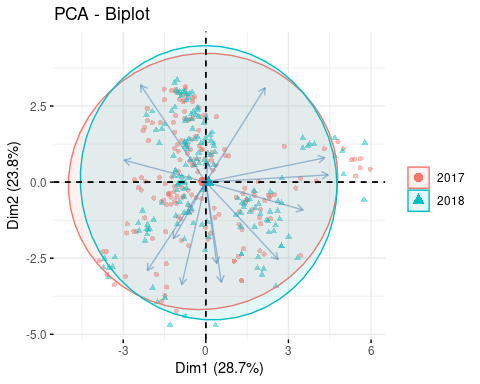 |
| --- | --- |

**Fig. S5** - Principal Component Analysis biplot of the measured soil physical-chemical parameters. AWC=Average water content. Org = Organic matter content.


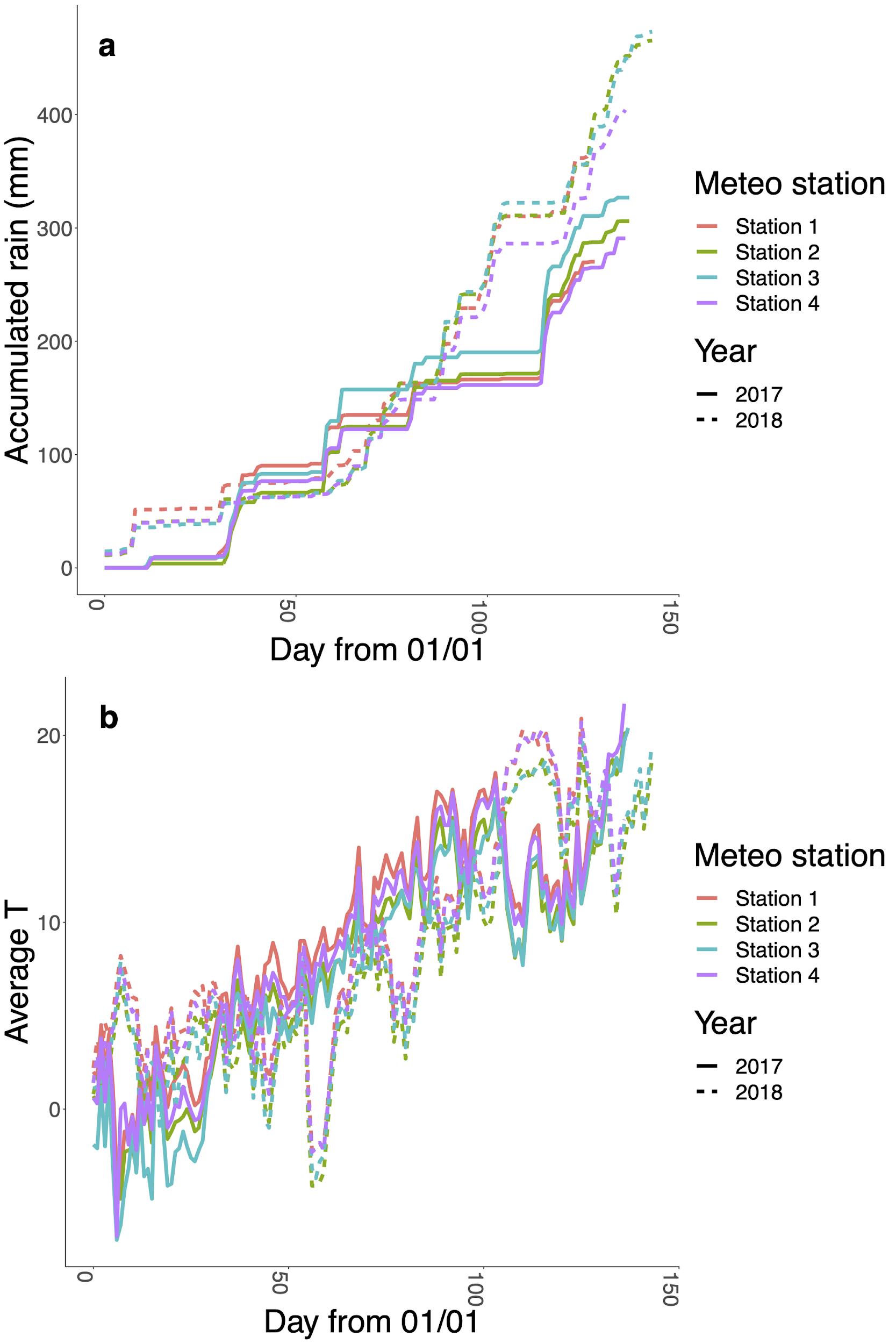


**Fig. S6** a) Accumulated amount of rain and b) mean daily temperature measured in four meteorological stations located close to the vineyards. Correspondence between the stations and the locations are as follows: Station 1 - Ala; Station2 -Besagno/Mori; Station 3 - S. Felice; Station 4 - Besagno. For each vineyard, soil sampling was performed on the last day of the series.

| 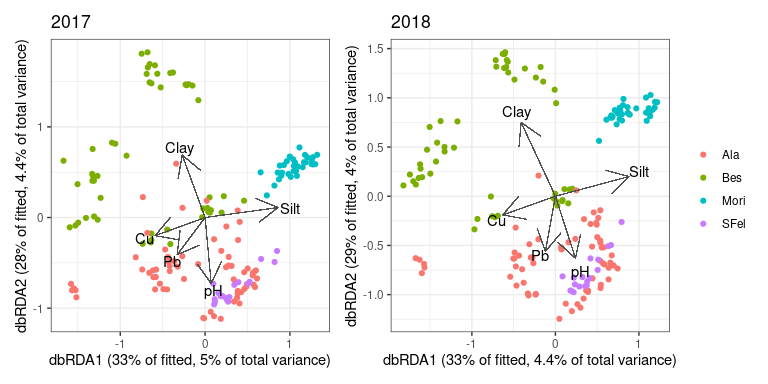 |
| --- |
| 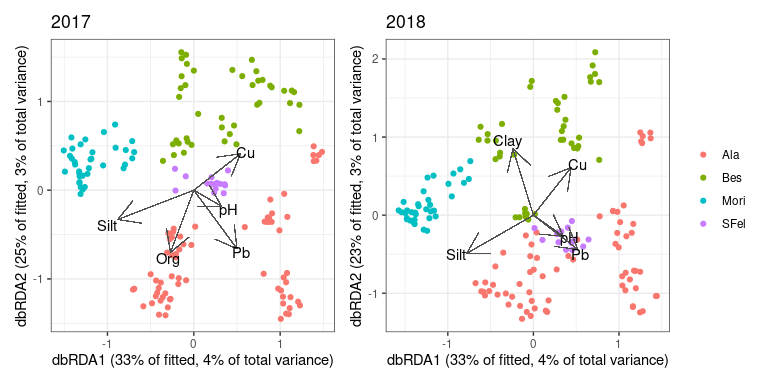 |

**Fig. S7** - dbRDA biplots for Bacteria & archea and Fungi. The top 5 environmental parameters were included as identified by the BioEnv procedure each year.


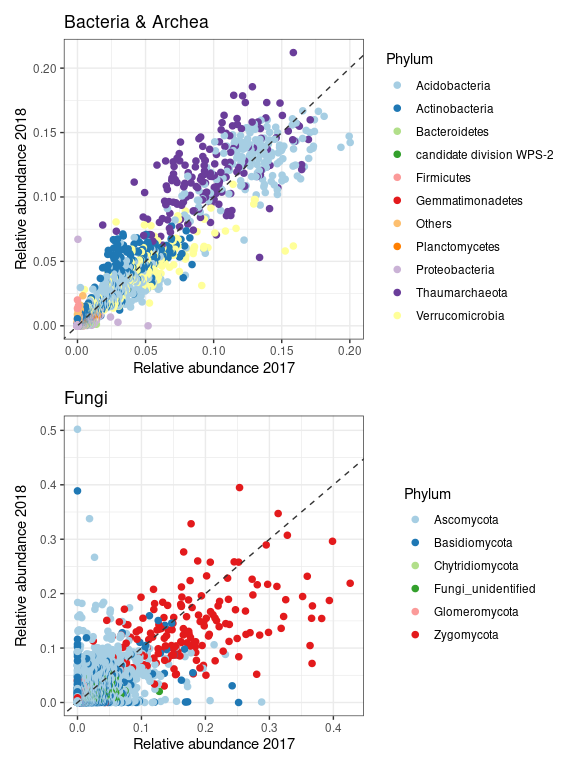


**Fig. S8** - Comparison of Genus relative abundance between the two years of study across all paired samples. Colours indicate the most abundant Phyla. Dashed line shows the 1:1 relationship.


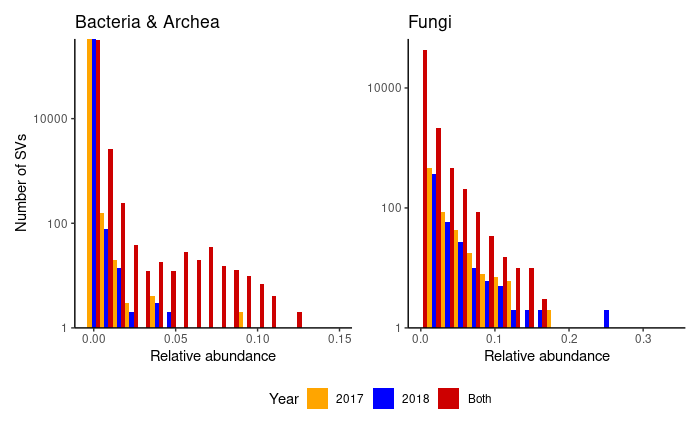


**Fig. S9** - Relative abundance of SVs that, within a given sample, were observed exclusively in one year or in both years.


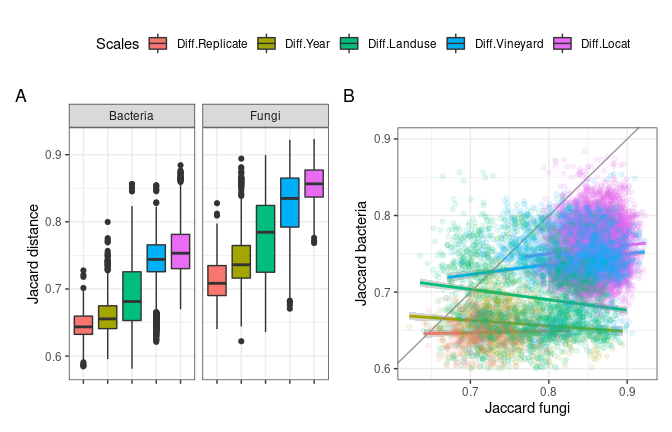


**Fig. S10** - A) pairwise Jaccard distance between samples for bacteria & archea and fungi across the scales of the study (i.e. between local replicates, years, land use, vineyards and locations). Each comparison is calculated by first ‘fixing’ the other scales. B) Relationship between fungal and bacterial pairwise Jaccard distances for each scale of the study. Thin grey line shows the hypothetical 1:1 relationship.

| Bacteria & Archea  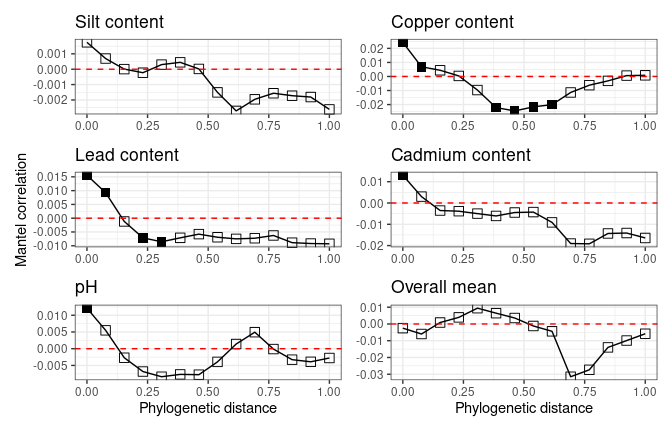 |
| --- |
| Fungi  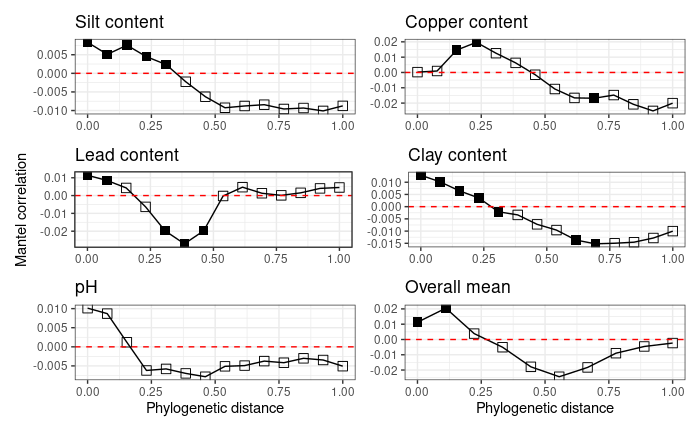 |

**Fig.S11** - Mantel correlograms between bacterial (top) and fungal (bottom) phylogenetic distance and niche distance based on soil abiotic variables. For a given variable, niche distance between taxa was measured as the absolute difference in their niche value, defined as the abundance-weighted mean of the environmental parameters where the species was present. Filled squares indicate significant Mantel correlation (p value < 0.05). Phylogenetic distances of bacteria and fungi were normalised to range between 0 and 1.
